# Etiología del maltrato a residentes de medicina desde la teoría de la violencia simbólica

**DOI:** 10.31053/1853.0605.v80.n3.42438

**Published:** 2023-09-29

**Authors:** Rosario Pérez Garcia, Manuela Pérez García

**Affiliations:** 1 Atención Primaria, Servicio Gallego de Salud (SERGAS) Santiago de Compostela España; 2 Servicio de Psiquiatría, Servicio Gallego de Salud (SERGAS) Santiago de Compostela España

Señor editor:

Queremos felicitar a Real y Ayala por su artículo "maltrato a residentes de medicina del Paraguay en 2022: estudio multicéntrico" por poner de relieve un tema que generalmente es silenciado, pero en el estudio se describe como un gran problema que afecta al 97,4% de los residentes encuestados ^1^ . Las condiciones laborales a las que los médicos residentes están sometidos vienen determinadas por la exigente formación médica recibida durante su residencia y por las condiciones adversas para el desempeño de su trabajo, como son la violencia física o verbal, la intimidación y el acoso laboral que deterioran y dificultan el ejercicio profesional afectando su salud física y emocional ^2^ . En este contexto hostil, el médico residente tiene que buscar su identidad profesional y enfrentarse a un contexto laboral generador de estrés y desigualdad ^3^ . En el informe de la OMS sobre la salud en el mundo se reporta que los profesionales sanitarios (médicos de pre y posgrado, médicos especialistas, enfermeras, entre otros) son proclives de sufrir actos de violencia interpersonal dentro de sus centros de trabajo, tanto por superiores como por compañeros y hasta por pacientes, siendo las principales afectadas del maltrato las profesionales sanitarias ^4^ .

La organización en los servicios de medicina hospitalaria se caracteriza por su jerarquización, donde la diferencia de conocimientos y de estatus entre directores médicos, jefes de servicio, médicos especialistas y residentes conlleva implícitamente una dinámica de poder generadora de maltrato. El tiempo de formación de los médicos residentes se caracteriza por la liminalidad, entendida como una fase de tránsito donde no son estudiantes de medicina, pero tampoco son médicos especialistas ya que todavía están formándose en sus respectivas especialidades. De este modo, los médicos residentes tienen un rol ambiguo y poco definido dentro del sistema de salud, generándoles estrés y malestar emocional ^5^ .

En una reciente revisión bibliográfica se evaluó la prevalencia del maltrato en residentes de medicina y cuáles fueron sus factores precipitantes. De la investigación se destaca que la prevalencia de maltrato entre los residentes fue del 51% lo que indica que existe una alta prevalencia de acoso entre los residentes a nivel global ^6^ . Los estudios incluidos en la revisión identificaron varios factores asociados con el maltrato, destacando que el ser mujer fue descrito como un factor de riesgo y los responsables del maltrato más comunes fueron los compañeros médicos residentes (66,7%), junto con los jefes de servicio (58,7%) ^2^ .

Dada su relevancia e implicaciones negativas para la salud mental de los residentes es necesario identificar las causas que promueven su elevada prevalencia a nivel global. Desde las teorías psicológicas se han propuesto diversas teorías para explicar la etiología del maltrato en general y de los médicos residentes en particular. La teoría de la disonancia cognitiva plantea un esclarecimiento de como los fenómenos psicológicos inconscientes que participan en la legitimación y justificación del maltrato por parte de la víctima y del victimario se perpetúan a lo largo del tiempo a pesar de la oposición social de la violencia gratuita ^7^ . Las teorías del aprendizaje vicario sustentan que se tiende a imitar el maltrato, a tal punto que se confunde si es imitación o miedo a ser maltratado, por lo que algunos terminan siendo quienes ejercen maltrato. Por ello, la violencia en todas sus manifestaciones, es consecuencia de comportamientos y actitudes aprendidos por imitación generando una dinámica por el cual la víctima se vuelve victimario ^8^ .

Para profundizar y ampliar nuestra comprensión de la etiología del maltrato nos valemos de las aportaciones de la teoría sobre la violencia simbólica descrita por Bourdieu ^9^ . Como ya se ha mencionado, el ámbito hospitalario está estructurado en jerarquías establecidas relacionadas con el poder que ostentan los médicos en los distintos estamentos. De tal suerte, que los de la jerarquía superior (directores médicos, jefe de servicio, médicos especialistas, médicos residentes en la finalización de su formación) disponen del poder que ocupan para dominar a los de jerarquía inferior (médicos residentes que inician su formación) que se encuentran en una posición de sumisión y de dependencia de la jerarquía.

Siguiendo a Bourdieu, el entramado que subyace al maltrato está formado por la violencia simbólica que se materializa en unas relaciones complejas en las que cada uno conoce su posición jerárquica y no la cuestiona ^9^ . Pero está violencia simbólica es experimentada de forma inconsciente, donde el maltrato se percibe como una actitud que forma parte de un orden establecido y normalizado donde el maltratador y el maltratado actúan sin cuestionar el maltrato y perpetuando con su comportamiento el modelo heredado históricamente ^9^ . Paradójicamente cuando el maltratado (residente médico que inician su formación) alcanza la categoría de especialista pasa a ser maltratador de los residentes que tienen a su cargo. De este modo, cuando un residente sube en la jerarquía, actúa con el mismo comportamiento que se espera de él reproduciendo las mismas las conductas hostiles que padeció. En relación al maltrato a las médicos residentes, se puede afirmar que la violencia simbólica legitima la violencia de género y del acoso sexual ya que viabiliza que sea percibida como parte inherente de la formación impartida por los maltratadores que ostentan puestos de poder en la jerarquía hospitalaria, que, en la mayoría de las ocasiones, son ocupados por hombres ^10^ .

Los responsables de las unidades docentes de residentes, las direcciones médicas hospitalarias, los jefes de servicio y demás profesionales médicos deben reflexionar sobre la necesidad de erradicar el maltrato a los residentes de medicina. Para ello, es preciso tomar conciencia de la fundamentación simbólica del maltrato que lo legitima y perpetua para desenmascarar la relación entre el maltratador y el maltratado, proponiendo un nuevo marco relacional basado en el respeto y el diálogo, para de este modo asumir las responsabilidades y las tareas que cada médico debe realizar en función de su cargo.
